# Real-Time Use of Monkeypox Virus Genomic Surveillance, King County, Washington, USA, 2022–2024

**DOI:** 10.3201/eid3113.241242

**Published:** 2025-05

**Authors:** Kathryn M. Lau, Michaela Banks, Kaila Bryant, Joanie D. Lambert, Laura Marcela Torres, Stephanie M. Lunn, Cory Yun, Pavitra Roychoudhury, B. Ethan Nunley, Jaydee Sereewit, Alexander L. Greninger, Allison Black, Vance Kawakami, Sargis Pogosjans, Elysia Gonzales, Eric J. Chow

**Affiliations:** Public Health–Seattle & King County, Seattle, Washington, USA (K.M. Lau, M. Banks, K. Bryant, J.D. Lambert, V. Kawakami, S. Pogosjans, E. Gonzales, E.J. Chow); Washington State Department of Health, Olympia, Washington, USA (L.M. Torres, S.M. Lunn, C. Yun, A. Black); University of Washington School of Medicine, Seattle (P. Roychoudhury, B.E. Nunley, J. Sereewit, A.L. Greninger, E.J. Chow); Fred Hutchinson Cancer Research Center, Seattle (P. Roychoudhury, A.L. Greninger); University of Washington School of Public Health, Seattle (E.J. Chow)

**Keywords:** Mpox, viruses, zoonoses, genomics, public health surveillance, monkeypox virus, whole-genome sequencing, molecular epidemiology, emerging communicable diseases, communicable disease control, public health practice, disease outbreaks, Washington, United States

## Abstract

A monkeypox virus genomic surveillance pilot began in King County, Washington, USA, during the 2022 outbreak. Genomic surveillance proved critical in determining local versus international exposure of a case where no known exposures were identified by interview, illustrating the value of genomics in case investigation and public health practice.

Mpox, an infectious disease caused by monkeypox virus (MPXV), emerged as a virus largely endemic to western and central Africa (*1*). When local transmission began to occur in many additional countries in 2022 (*1*), whole-genome sequencing (WGS) of MPXV offered a potential new tool to complement traditional case investigations and identify epidemiologic linkages. Public Health–Seattle & King County (PHSKC), Washington State Department of Health (WADOH), and the University of Washington Virology Laboratory (UW Virology) collaborated to pilot a retrospective genomic surveillance program investigating mpox in King County, Washington, USA, in September 2022. PHSKC subsequently used retrospective data from the pilot and real-time WGS to support investigation of a case of mpox with unknown exposure.

Data were collected as part of routine public health surveillance and are considered nonresearch. Patient consent was not required, but verbal consent was obtained from the patient whose case is described, and all identifying details of the patient have been removed in accordance with the institutional policy of PHSKC.

## Methods

In September 2022, WADOH and PHSKC retrospectively linked WGS and epidemiologic data for cases of mpox occurring since May 2022 in King County. PHSKC collected epidemiologic data using case interviews and chart reviews for every reported case of mpox. UW Virology generated sequences and attempted WGS on mpox-positive residual diagnostic specimens using a hybridization probe-capture–based approach with probes designed using the MPXV 2022/MA001 strain (*2*). Laboratory staff generated consensus genomes using a custom Nextflow pipeline (https://github.com/greninger-lab/nf_mpxv_f13l). WADOH built a phylogenetic tree of all local cases and contextual sequences from other regions (*3*), and PHSKC annotated the tree with epidemiologic data. During September 2022–July 2024, PHSKC actively pursued WGS of all new mpox cases, linked WGS results to cases, and analyzed phylogenetic relatedness using Nextstrain and Nextclade (*4*,*5*).

## Results

The retrospective analysis linked WGS results to 126 mpox cases that occurred during May–September 2022 (29.6% of 426 total cases during the pilot period). All isolates belonged to clade IIb, lineage B.1—the lineage identified in most sequences from the 2022–2023 global outbreak (*1*,*6*).

In fall 2023, an adult man residing in King County, Washington, had mpox symptoms develop 2 days after a 5-day trip to Kenya with an overnight layover in United Arab Emirates (UAE). Symptoms began with small, blister-like penile lesions. Five days after lesions appeared, the man developed headache, groin pain, fatigue, and subjective fever. Eight days after symptom onset, healthcare professionals collected a lesion specimen, which tested positive for mpox by real-time PCR. The man completed a prescribed course of oral tecovirimat and reported fever and pain resolution 18 days after symptom onset and complete scab resolution 24 days after symptom onset. On the basis of symptom onset date and a typical incubation period of 3–17 days, the exposure period spanned dates when the man was in King County, Kenya, and the UAE (*7*). During case interviews, he reported having no exposure to anyone with known or suspected mpox and no sexual activity or close physical contact with anyone 3 weeks before symptom onset. According to interview alone, investigators remained uncertain where and when the man was likely exposed.

During September 2022–fall 2023, 71 cases from Washington were sequenced in realtime (37.4% of 190 total cases), and all real-time sequenced cases were lineage B.1. WGS results for this case were shared with PHSKC 15 days after the case report, and the sequence was identified as Clade IIb, sublineage A.2.1. The sequence was highly divergent from lineage B.1, showing ≈94 nt variations (Figure). Although lineage B.1 was identified in most sequences in the 2022–2023 outbreak, including in local transmission in the United States, lineage A.2 had been detected infrequently outside of endemic areas (*1*,*6*). A published analysis of the 3 previous mpox cases in the United States with lineage A.2 concluded each was likely a separate introduction, with suspected exposure during travel to the Middle East or West Africa (*6*). The high divergence from other Washington cases and the rarity of reported A.2 lineages in the United States strongly suggested that this case-patient’s exposure occurred during international travel.

**Figure:**
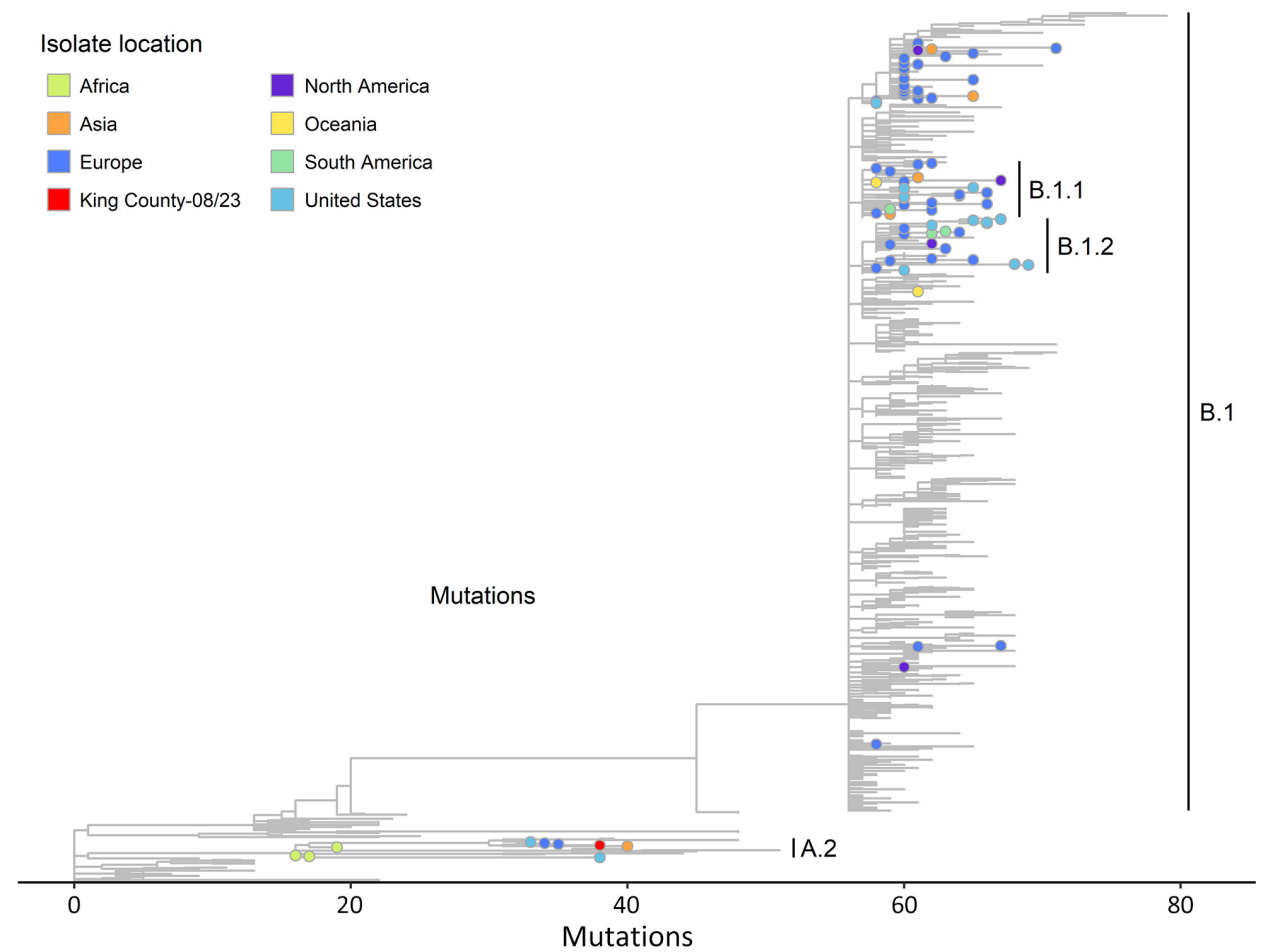
Phylogenetic reference tree of clade IIb monkeypox virus sequences as of December 2023 from study of real-time use of monkeypox virus genomic surveillance, King County, Washington, USA, 2022–2024. The tree shows the viral strain of sublineage A.2.1 identified from a King County, resident in fall 2023 (in red) is highly diverged from the B.1 lineage. Colors correspond to the location of the case from which a viral isolate was sampled and are either a region, the United States, or King County, for the specific isolate of interest in this case report. Phylogenetic tree generated using Nextclade dataset for “Mpox virus (All Clades)” in Auspice v2.61.1 (https://github.com/nextstrain/auspice.us). The reference sequence used in the tree is the clade IIb GenBank reference sequence (accession no. NC_063383.1). Data sourced from GenBank on December 8, 2023 (*13*). Sequences were downsampled by the Nextstrain team to ≈500 sequences with the goal of capturing monkeypox virus diversity across geography, collection dates, and lineages. The dataset is archived by Nextclade (https://github.com/nextstrain/Nextclade_data/tree/master/data_output/nextstrain/mpox/all-clades/2024-01-16--20-31-02Z). The figure is filtered to the clade IIb branch within the larger dataset. Branches are shown for all lineages within clade IIb, and specific nodes are shown for selected lineages A.2, A.2.1, B.1.1, B.1.2, and B.1.3. A table of the nodes with metadata is included in the Appendix.

As of fall 2023, there were zero cases of mpox reported in Kenya and 16 in the UAE; lineage A.2 specifically was reported among at least nine travelers returning from the UAE in 2022 (*8*–*11*), strongly suggesting exposure in the UAE. During fall 2023–July 2024, 37 cases of mpox were reported in King County, and 76% of the cases had samples that were sequenced (n = 28). All sequences were lineage B.1, suggesting no onward transmission of lineage A.2 locally.

## Discussion

This case illustrates the value of genomic surveillance in mpox public health response. No known exposures could be identified during case investigation through patient interview, but sequencing helped public health staff determine that this case was unlikely representative of undetected local spread, which reduced concern regarding additional, unreported cases. Applying WGS in practice required close collaboration across agencies to proactively establish local genomic diversity and conduct active genomic surveillance. The 2022–2023 mpox outbreak disproportionately affected men who have sex with men, and interview participation could have been limited by concerns about stigma (*12*). Genomic surveillance has potential to answer broad questions of concern for public health, like whether local transmission is occurring, without requiring detailed exposure information. As use of pathogen sequencing expands, additional work should be conducted to anticipate potential ethical concerns and establish practices that protect privacy while advancing infectious disease prevention and response.

Appendix Additional information for real-time use of mpox genomic surveillance, King County, Washington, USA, 2022–2024.
